# *Deinococcus radiodurans*-derived membrane vesicles protect HaCaT cells against H_2_O_2_-induced oxidative stress via modulation of MAPK and Nrf2/ARE pathways

**DOI:** 10.1186/s12575-023-00211-4

**Published:** 2023-06-16

**Authors:** Jeong Moo Han, Ha-Yeon Song, Jong-Hyun Jung, Sangyong Lim, Ho Seong Seo, Woo Sik Kim, Seung-Taik Lim, Eui-Baek Byun

**Affiliations:** 1 Advanced Radiation Technology Institute, Korea Atomic Energy Research Institute, Jeongeup-Si, Jeollabuk-Do 56212 Republic of Korea; 2 Department of Biotechnology, College of Life Science and Biotechnology, Korea University, Seoul, 136-701 Republic of Korea; 3 Department of Radiation Science, University of Science and Technology, Daejeon, 34113 Republic of Korea; 4 Functional Biomaterial Research Center, Korea Research Institute of Bioscience and Biotechnology, Jeongeup-Si, Jeollabuk-Do 56212 Republic of Korea

**Keywords:** *Deinococcus radiodurans*, ΔDR2577 mutant, Extracellular vesicles, Oxidative stress, Antioxidant

## Abstract

**Background:**

*Deinococcus radiodurans* is a robust bacterium that can withstand harsh environments that cause oxidative stress to macromolecules due to its cellular structure and physiological functions. Cells release extracellular vesicles for intercellular communication and the transfer of biological information; their payload reflects the status of the source cells. Yet, the biological role and mechanism of *Deinococcus radiodurans*-derived extracellular vesicles remain unclear.

**Aim:**

This study investigated the protective effects of membrane vesicles derived from *D. radiodurans* (R1-MVs) against H_2_O_2_-induced oxidative stress in HaCaT cells.

**Results:**

R1-MVs were identified as 322 nm spherical molecules. Pretreatment with R1-MVs inhibited H_2_O_2_-mediated apoptosis in HaCaT cells by suppressing the loss of mitochondrial membrane potential and reactive oxygen species (ROS) production. R1-MVs increased the superoxide dismutase (SOD) and catalase (CAT) activities, restored glutathione (GSH) homeostasis, and reduced malondialdehyde (MDA) production in H_2_O_2_-exposed HaCaT cells. Moreover, the protective effect of R1-MVs against H_2_O_2_-induced oxidative stress in HaCaT cells was dependent on the downregulation of mitogen-activated protein kinase (MAPK) phosphorylation and the upregulation of the nuclear factor E2-related factor 2 (Nrf2)/antioxidant response element (ARE) pathway. Furthermore, the weaker protective capabilities of R1-MVs derived from ΔDR2577 mutant than that of the wild-type R1-MVs confirmed our inferences and indicated that SlpA protein plays a crucial role in R1-MVs against H_2_O_2_-induced oxidative stress.

**Conclusion:**

Taken together, R1-MVs exert significant protective effects against H_2_O_2_-induced oxidative stress in keratinocytes and have the potential to be applied in radiation-induced oxidative stress models.

**Supplementary Information:**

The online version contains supplementary material available at 10.1186/s12575-023-00211-4.

## Background

*Deinococcus radiodurans* is an extremophilic bacterium well known for its high level of resistance to ionizing radiation [1]. *Deinococcus radiodurans* has evolved extremely effective radiation and oxidative stress protection systems, including passive and active defense mechanisms [2]. The genome of *D. radiodurans* is densely packaged and forms nucleoid with toroidal architecture, which may shield DNA from radiation and oxidative stress. In addition, Deinococcus-specific proteins, such as DdrB and IrrE/DdrO were involved in the repair and protection of DNA and protein [3, 4]. Importantly, *D. radiodurans* can protect against oxidative stress that damages proteins, nucleic acids, and lipids by mediating effective redox homeostasis and scavenging reactive oxygen species (ROS) [5]. The cellular ROS-scavenging capacity of *D. radiodurans* comprises antioxidant enzymes, including superoxide dismutase (SOD), catalase (CAT), peroxidase, peroxiredoxins, and thioredoxin, and non-enzymatic antioxidants, such as accumulated Mn^2+^, pyrroloquinoline–quinone, and deinoxanthin [6]. Based on these characteristics of *D. radiodurans*, various studies have explored its potential for biomedicine. For example, deinoxanthin, a xanthophyll derived from *Deinococcus* species, has attracted considerable attention because of its significant antioxidant effects and ROS scavenging activity [7, 8]. Exopolysaccharides produced by *D. radiodurans* have been reported to scavenge H_2_O_2_ and exert anti-apoptotic effects in human keratinocytes [9].

Membrane vesicles (MV) are lipid bilayer membrane-enclosed nanovesicles produced by most bacteria, along with various biomolecules, such as proteins (including enzymes and transcriptional factors), lipids, nucleic acids, and metabolites [10, 11]. Bacterial MVs are secreted from the cell surface into the extracellular space and are called extracellular vesicles (EVs) [12]. EVs play crucial roles in intercellular communication by transferring bioactive components from donor to recipient cells and mediating various physiological and pathological processes, including molecular transport, stress response mediation, and interaction with the host [13, 14]. EVs derived from pathogenic bacteria play a pathological role by delivering virulence factors and toxins to target cells [15]. In contrast, gut microbiota and probiotic-derived EVs induce fortification of the gut barrier and suppress inflammation [16]. EVs mirror the physiological state of the parent cells and are used to deliver messages [17]. Therefore, EVs have been highlighted as potential diagnostic and therapeutic agents that can be applied for various biological processes, such as tissue signaling [18], immune modulation [19], metastasis spreading [20], and wound healing [21].

Although *D. radiodurans* is extremely resistant to oxidative stress [5], the functionality of MVs derived from *D. radiodurans* has not been characterized*.* This study aimed to isolate and characterize *D. radiodurans*-derived MVs (R1-MVsMVs) and to identify the antioxidative properties of R1-MVsMVs against H_2_O_2_-induced oxidative stress in human keratinocytes.

## Results

### Isolation and characterization of MVs derived from *D. radiodurans*

**Fig. 1:**
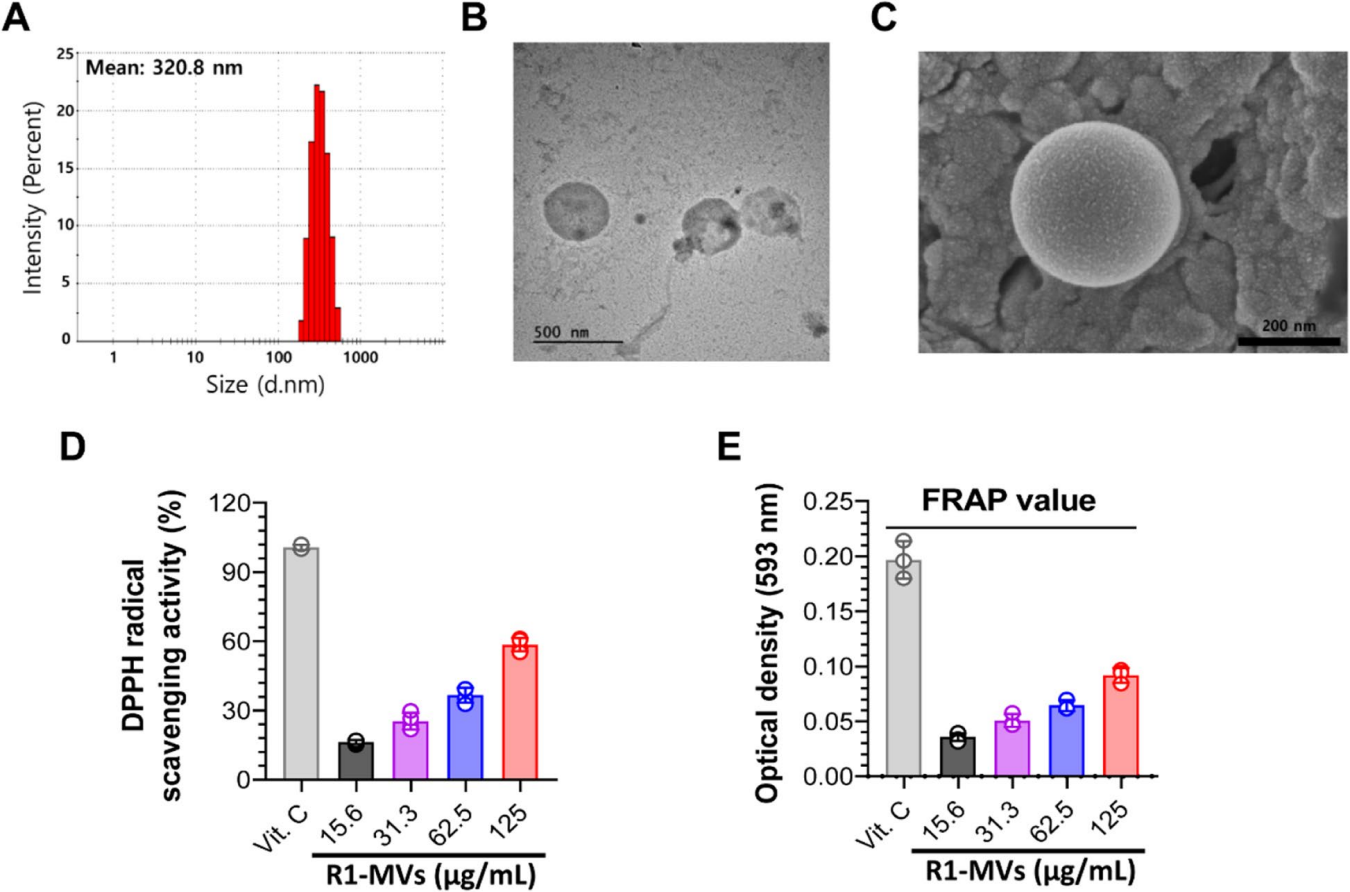
Isolation and characterization of extracellular vesicles (R1-MVs) derived from *Deinococcus radiodurans.* **A** Size distribution of R1-MVs determined using dynamic light scattering (DLS) analysis. **B** Morphology of R1-MVs visualized by transmission electron microscopy (TEM). Scale bar = 500 nm. **C** Visualization of R1-MVs using scanning electron microscopy (SEM). Scale bar = 200 nm. **D** and **E** The antioxidant activity of R1-MVs analyzed using (**D**) DPPH and (**E**) FRAP assays

R1-MVsMVs were isolated from cultured *D. radiodurans* supernatants via filtration and differential centrifugation. R1-MVs had an average diameter of 300 nm as determined by DLS analysis (Fig. 1A). In agreement with the DLS results, TEM and SEM analyses confirmed that the size of the R1-MVs ranged from 290 to 330 nm (Fig. 1B and C). Next, we confirmed the antioxidant activities of R1-MVs using DPPH and FRAP assay that R1-MVs (0.25–2 mg/mL) induced radical scavenging and ferric-reducing abilities in a dose-dependent manner (Fig. 1D and E). These results suggested that R1-MVs have potential, direct and indirect, protective roles against oxidative stress.

### R1-MVs inhibit H_2_O_2_ -induced cytotoxicity in HaCaT cells

**Fig. 2:**
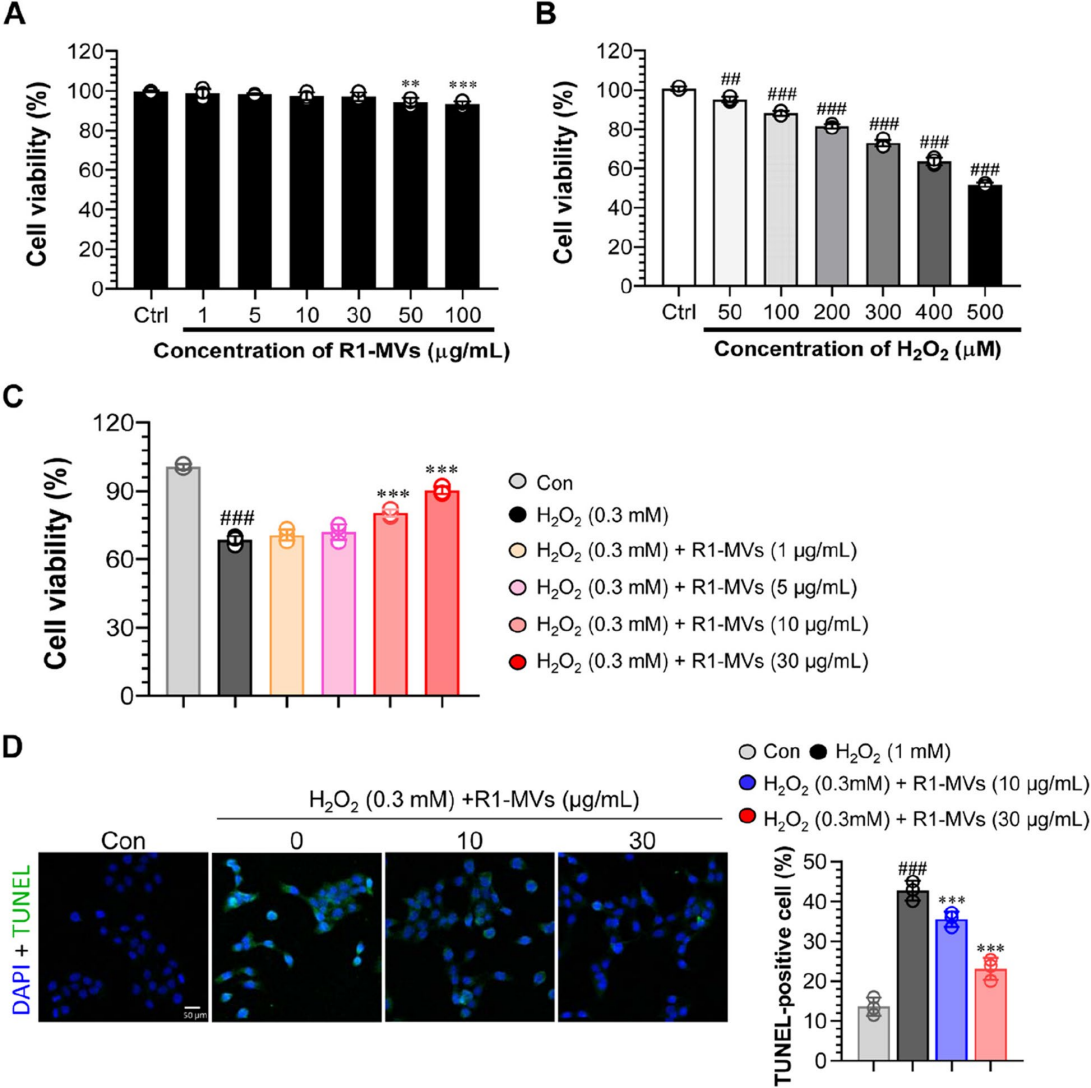
Effects of R1-MVs on H_2_O_2_-induced HaCaT cell damage. **A** Cytotoxicity of R1-MVs (1, 5, 10, 30, 50 and 100 μg/mL) on HaCaT cells analyzed using MTT assay. **B** The cells were exposed to H_2_O_2_ (50, 100, 200, 300, 400, and 500 μM) for 12 h, and the cell viability was assessed using MTT assay to assess the cytotoxicity and optimal dose of H_2_O_2_. **C** HaCaT cells were pretreated with different concentrations of R1-MVs (1, 5, 10, and 30 μg/mL) for 12 h before exposure to H_2_O_2_ (0.3 mM) for 12 h, and the cell viability was measured using MTT assay to assess the cytotoxicity and optimal dose of R1-MVs. **D** HaCaT cells were stained with TUNEL and examined using a 20 × fluorescence microscope to assess inhibition of cell death by R1-MVs. Scale bar: 50 μm. The fluorescence quantitative analysis using Image J software. The data show the mean ± SD (*n* = 4 samples) of three representative experiments. ^##^*p* < 0.01 or ^###^*p* < 0.001 vs. control group; *p < 0.05, **p < 0.01, or ***p < 0.001 vs. H_2_O_2_-treated group

Evaluation of the cytotoxicity of R1-MVs in HaCaT cells using the MTT assay demonstrated that treatment with R1-MVs for 18 h was not cytotoxic to HaCaT cells at concentrations of up to 30 μg/mL (Fig. 2A). Furthermore, exposure of HaCaT cells to different concentrations of H_2_O_2_ (50, 100, 200, 300, 400, and 500 μM) showed that H_2_O_2_ at 300 μM decreased cell viability to 70% compared to that of the untreated group. Therefore, this concentration was used as the optimal dose to induce damage for all subsequent experiments (Fig. 2B). Pretreatment of HaCaT cells with the R1-MVs followed by H_2_O_2_ treatment revealed that R1-MVs at 10 or 30 μg/mL increased viability of H_2_O_2_-treated HaCaT cells dose-dependently (Fig. 2C). The protective effect of R1-MVs in H_2_O_2_-treated HaCaT cells was confirmed by morphological assessment using TUNEL staining. H_2_O_2_-treated HaCaT cells showed severe nuclear fragmentation; however, pretreatment with R1-MVs at 10 or 30 μg/mL induced the inhibition of nuclear fragmentation (Fig. 2D). Therefore, R1-MVs have the potential to the protect against oxidative stress. The data show the mean ± SD (*n* = 3 samples) of three representative experiments.

### Effects of R1-MVs on the production of intracellular ROS

**Fig. 3:**
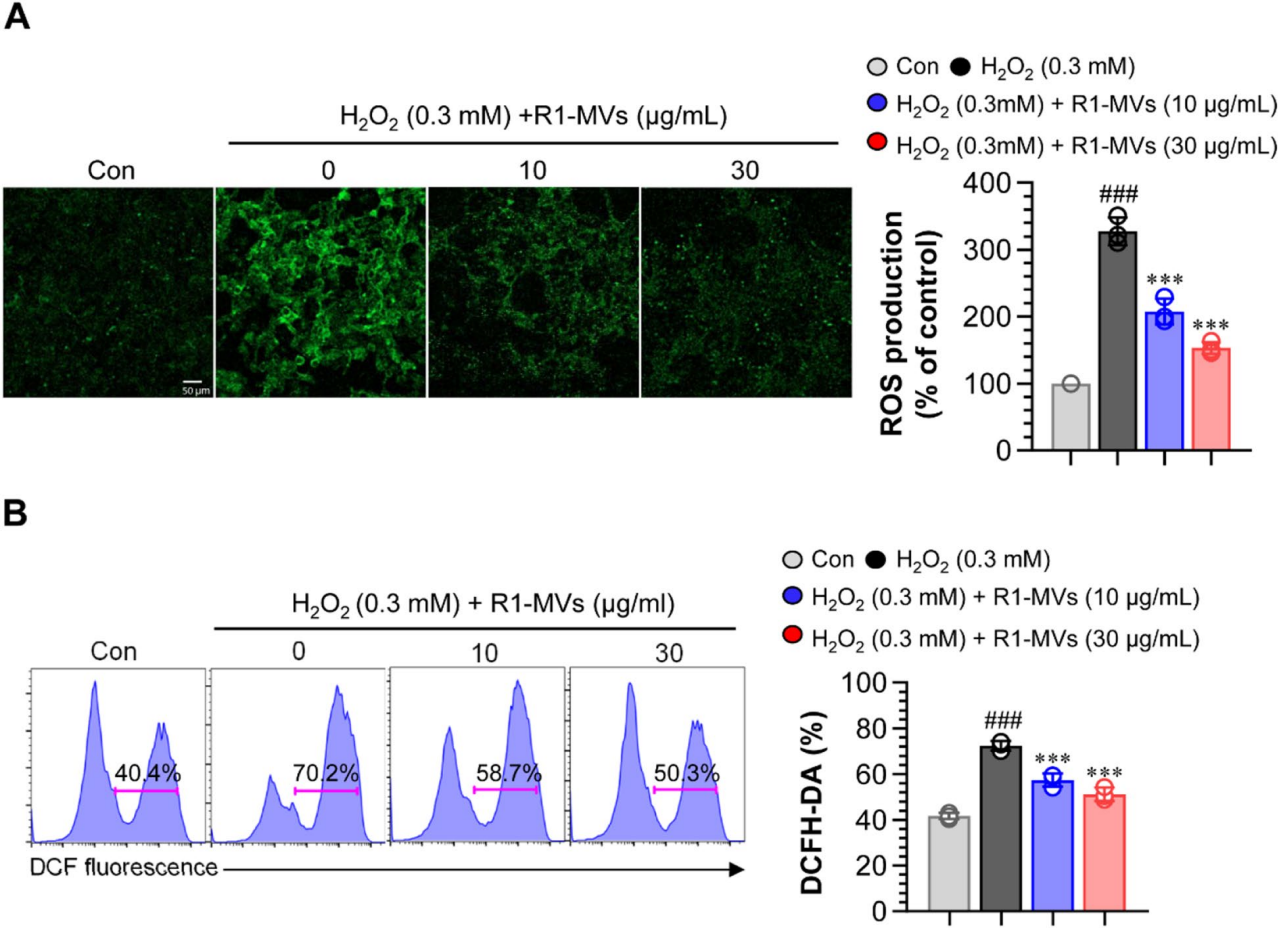
Effects of R1-MVs on intracellular ROS (fluorescence intensity) in H_2_O_2_-treated HaCaT cells. Cells were pretreated with different concentrations of R1-MVs for 12 h followed by treatment with 0.3 mM H_2_O_2_ for 12 h. **A** Intracellular ROS level detected by DCFH-DA using fluorescence microscopy and quantitative analysis using ImageJ software. Scale bar: 50 μm. **B** Intracellular ROS level measured by DCFH-DA using flow cytometry. ^###^*p* < 0.001 vs. control group; *p < 0.05, **p < 0.01, or ***p < 0.001 vs. H_2_O_2_-treated group. The data show the mean ± SD (*n* = 4 samples) of three representative experiments

To identify the mechanism underlying the protective effect of R1-MVs against H_2_O_2_, intracellular levels of ROS were assessed using DCF-DA. Endogenous ROS levels were assessed using confocal microscopy (Fig. 3A) and flow cytometry (Fig. 3B). Fluorescence intensity of the HaCaT cells exposed to H_2_O_2_ revealed a higher production of intracellular ROS than that in the control group. However, treatment with R1-MVs (10 and 30 μg/mL) prior to H_2_O_2_ treatment considerably reduced the production of intracellular ROS (Fig. 3A and B). These results suggest that R1-MVs inhibit the production and accumulation of intracellular ROS and alleviate H_2_O_2_-induced oxidative stress in HaCaT cells.

### Effects of R1-MVs on MDA content, SOD and CAT activities, and GSH level

**Fig. 4:**
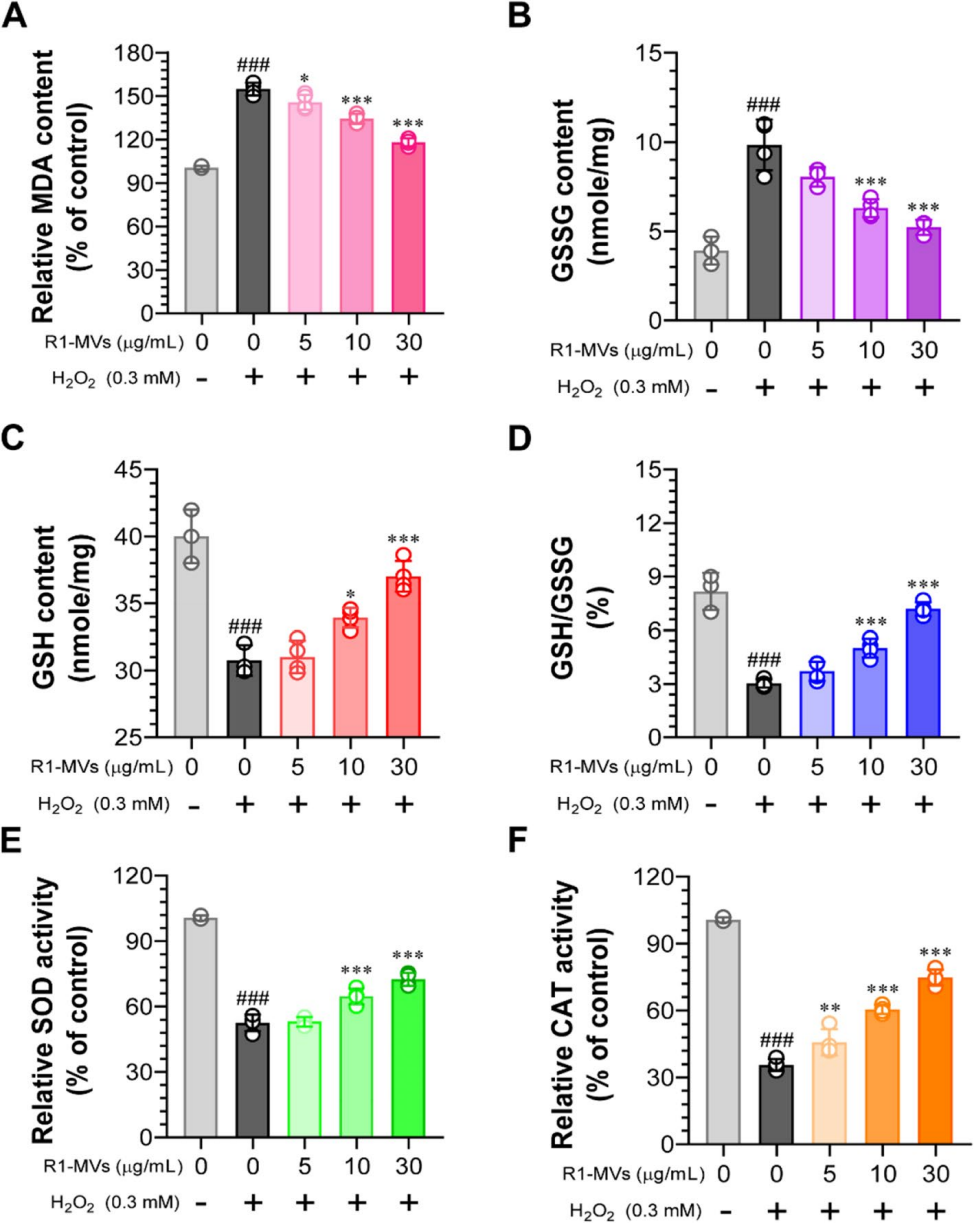
Effect of R1-MVs on the activities of antioxidant enzymes (SOD and CAT), level of GSH, and MDA content in H_2_O_2_-treated HaCaT cells. Cells were pretreated with different concentrations of R1-MVs (5, 10, and 30 μg/mL) for 12 h, followed by treatment with 0.3 mM H_2_O_2_ for 12 h. **A** Malondialdehyde (MDA) content, (**B**) oxidized glutathione (GSSG) content, (**C**) glutathione (GSH) content, (**D**) GSH/GSSG ratio, (**E**) superoxide dismutase (SOD) activity, and (**F**) catalase (CAT) activity assessed in HaCaT cells. ^###^*p* < 0.001 vs. control group; *p < 0.05, **p < 0.01, or ***p < 0.001 vs. H_2_O_2_-treated group. The data show the mean ± SD (*n* = 4 samples) of three representative experiments

ROS overproduction can lead to lipid peroxidation, and MDA is a biomarker of lipid peroxidation [22]. Considering the mechanism of lipid peroxidation, we evaluated the effect of R1-MVs on H_2_O_2_-induced MDA overproduction in HaCaT cells. As shown in Fig. 4A, the MDA content was significantly increased after H_2_O_2_ treatment compared to that in the control group. Compared to the H_2_O_2_-treated group, R1-MVs (5, 10, and 30 μg/mL) pretreatment inhibited the increase in MDA content upon exposure to H_2_O_2_ in HaCaT cells. Furthermore, to determine whether R1-MVs could inhibit H_2_O_2_-induced oxidative stress by regulating the intracellular antioxidant system, the activities of SOD and CAT and the levels of GSH were measured [23–25]. H_2_O_2_ alone-treated HaCaT cells significantly increased oxidized glutathione (GSSG) content and decreased GSH, SOD, and CAT levels compared to the control group. However, pretreatment with R1-MVs (10 and 30 μg/mL) considerably restored GSH homeostasis (increased GSH/GSSG ratio) and increased the activities of SOD and CAT (Fig. 4B–F). These results suggest that R1-MVs reduce MDA content and play an important role in maintaining membranes by suppressing lipid peroxidation. In addition, R1-MVs can effectively enhance antioxidant-related molecules, including the activities of SOD and CAT and the level of GSH.

### Effects of R1-MVs on mitochondrial membrane potential and apoptotic pathways in HaCaT cells

**Fig. 5:**
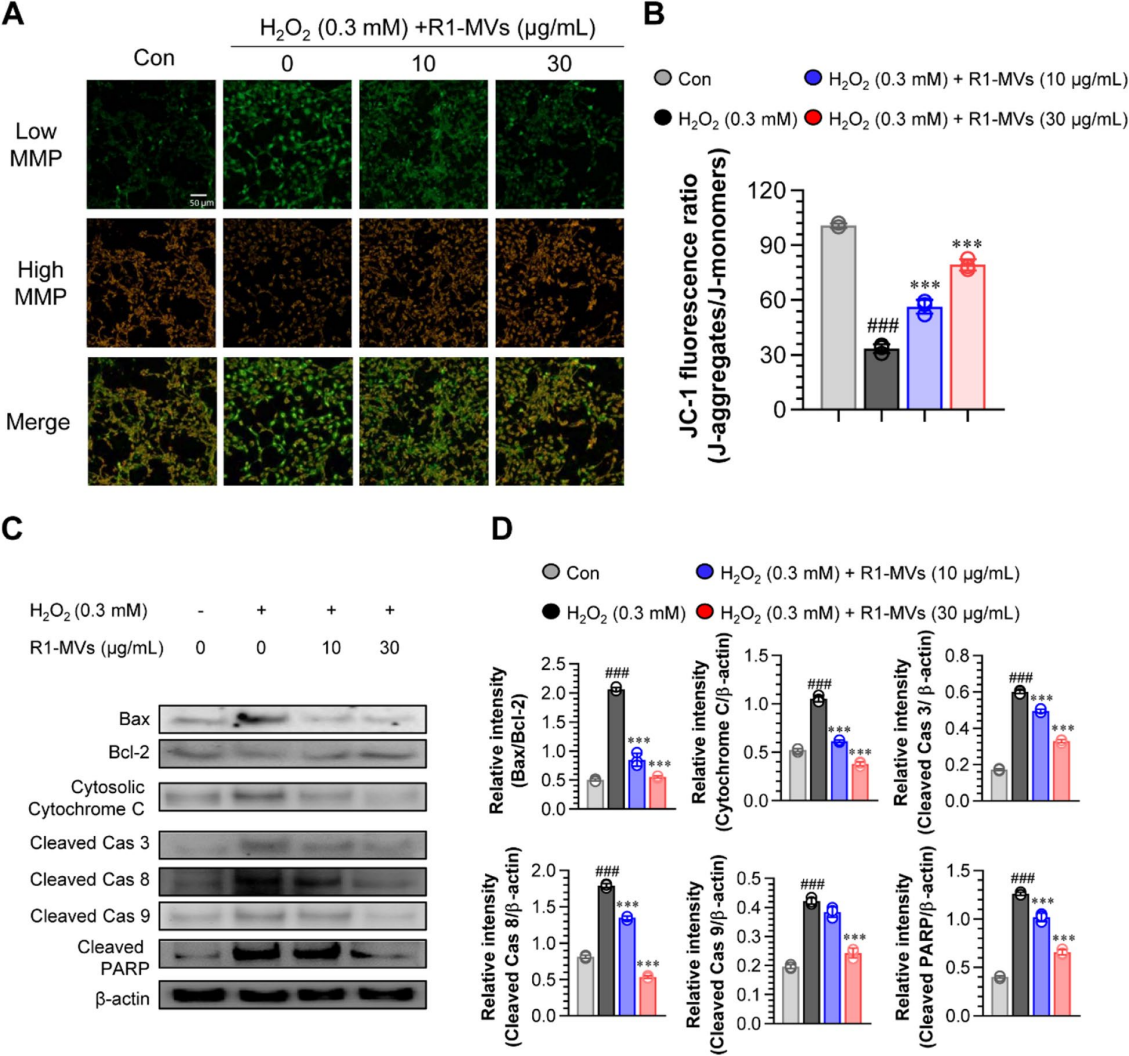
Effect of R1-MVs on MMP and expression of apoptotic proteins in H_2_O_2_-treated HaCaT cells. HaCaT cells were pre-processed with R1-MVs at different concentrations (10 and 30 μg/mL) for 12 h prior to exposure to H_2_O_2_ (0.3 mM) for 12 h. **A** The change in MMP was detected by JC-1 using a 20 × laser scanning confocal microscope. Scale bar: 50 nm. **B** Percentage of fluorescence intensity specific value of the red/green quantified by ImageJ software. Significance: ^###^*p* < 0.001 vs. control; *p < 0.05 and **p < 0.01 vs. H_2_O_2_-treated group. **C** Expression of proteins related to apoptosis signals using specific antibodies against Bax, Bcl-2, Cytochrome C (cytosol), Cleaved Caspase 3, Cleaved Caspase 8, Cleaved Caspase 9, Cleaved poly (ADP-ribose) polymerase (PARP), and β-actin. **D** The relative band intensity of each protein evaluated using ImageJ software and expressed as a percentage. ^###^*p* < 0.001 vs. control group; *p < 0.05, **p < 0.01, or ***p < 0.001 vs. H_2_O_2_-treated group. The values are mean ± SD (*n* = 4 samples) of three representative experiments

Excessive ROS exposure will disrupt the redox homeostasis, leading to oxidative stress and ROS-mediated damage of biomolecules such as DNA and proteins as well as organelles including mitochondria. Increased cellular ROS levels induce the loss of MMP, a landmark in early apoptosis [26]. In healthy cells with a normal MMP, the JC-1 dye enters and forms red fluorescent J-aggregates. Meanwhile, in apoptotic cells, the JC-dye cannot penetrate the cells to induce the formation of J-aggregates and presents with green fluorescence instead [27]. Here, we assessed the ability of R1-MVs to repress the MMP drop induced by H_2_O_2_ using a mitochondrial-specific JC-1 probe [28]. The H_2_O_2_-treated group showed a decreased red and increased green fluorescence, indicating increased apoptosis. In contrast, pretreatment with R1-MVs (10 and 30 μg/mL) significantly increased red fluorescence and decreased green fluorescence, indicating a reduction in apoptotic cells in H_2_O_2_-treated HaCaT cells (Fig. 5A and B). Since excessive ROS production induced cell death, inhibition of ROS-induced apoptosis is important for maintaining cellular homeostasis [29, 30]. To investigate whether pretreatment with R1-MVs suppressed the activation of the apoptosis signaling pathway, the expression levels of various apoptotic indicators, including pro-apoptotic (BAX) and anti-apoptotic (BCL-2) proteins, as well as cytochrome c, cleaved poly (ADP-ribose) polymerase (PARP), and cleaved caspases-3, -8, and -9 in H_2_O_2_-treated HaCaT cells were measured. The results showed that exposure to H_2_O_2_ elevated the expression of BAX and cytosolic cytochrome c, decreased the levels of BCL-2, and increased the cleavage of PARP and cleaved caspase (cleaved caspases-3, -8, and -9) in HaCaT cells. However, pretreatment with R1-MVs (10 and 30 μg/mL) before exposure to H_2_O_2_ significantly inhibited the upregulation of BAX, cytosolic cytochrome c, and caspases-3, -8, and -9, as well as PARP cleavage and downregulation of BCL-2 in a concentration-dependent manner (Fig. 5C and D). Taken together, R1-MVs exert an inhibitory effect on MMP loss and the apoptotic cascade cell death in H_2_O_2_-induced oxidative stress in HaCaT cells.

### Effects of R1-MVs on MAPK signaling pathways associated with oxidative stress

**Fig. 6:**
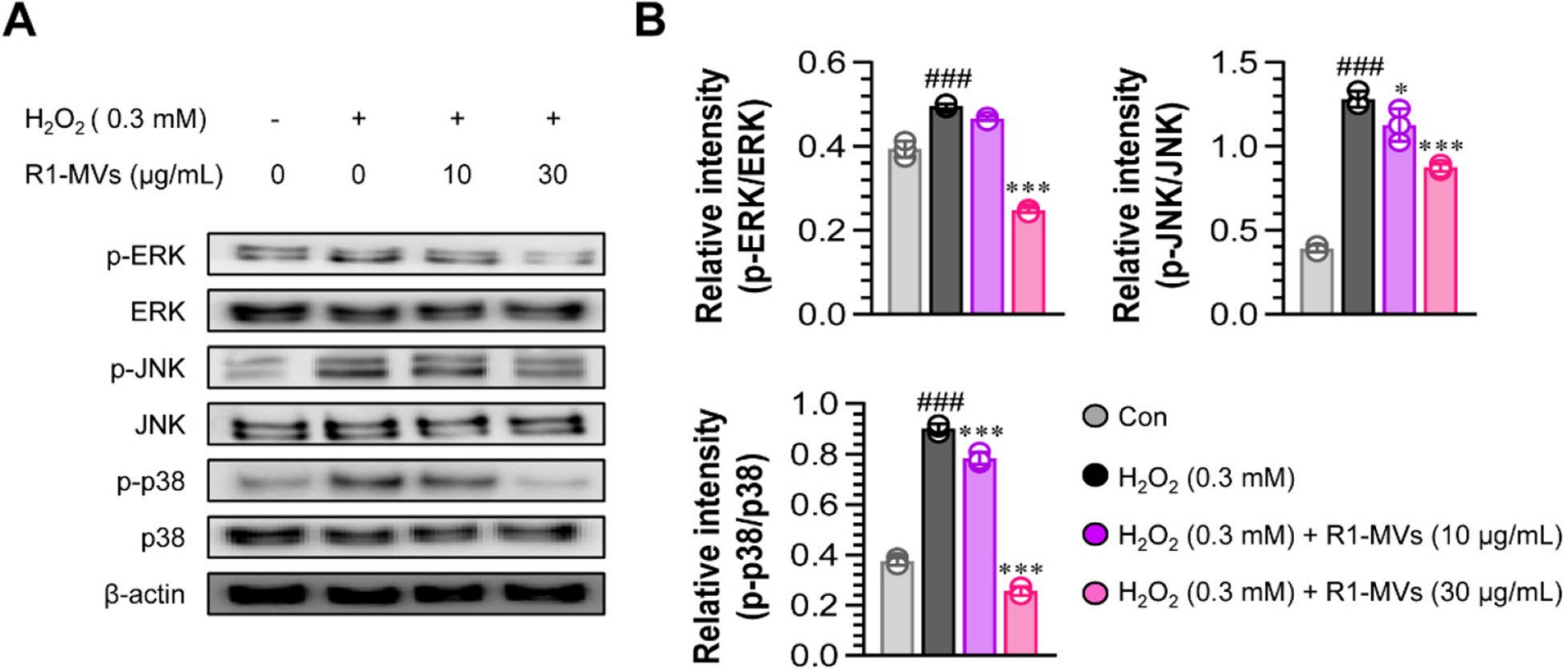
Effects of R1-MVs on the activation of the MAPK pathway in H_2_O_2_-treated HaCaT cells. HaCaT cells were pre-processed with R1-MVs at different concentrations (10 and 30 μg/mL) for 12 h prior to exposure to H_2_O_2_ (0.3 mM) for 12 h. **A** The expression of phosphor-p38 (p-p38), p38, p-ERK, ERK, p-JNK, JNK, and β-actin was measured using western blotting analysis. **B** The relative MAPK band intensity of each protein evaluated using ImageJ is expressed as a percentage. ^###^*p* < 0.001 vs. control group; *p < 0.05, **p < 0.01, or ***p < 0.001 vs. H_2_O_2_-treated group. The data show the mean ± SD (*n* = 4 samples) of three representative experiments

Excessive ROS generation induced by H_2_O_2_ can activate mitogen-activated protein kinase (MAPK) signaling pathways [31]. MAPK pathways are activated by external stimulation and are related to the immune response, inflammation, and apoptosis [32]. To determine whether the protective effect of R1-MVs in HaCaT cells exposed to H_2_O_2_ occurs via the regulation of the MAPKs pathway, we evaluated the presence and absence of R1-MVs in H_2_O_2_-stimulated HaCaT cells. The results indicated that the phosphorylation of MAPKs (p38, ERK, and JNK) proteins induced by H_2_O_2_ was decreased by pretreatment with R1-MVs (10 and 30 μg/mL) compared to H_2_O_2_ alone-treated HaCaT cells (Fig. 6A and B). These results suggest that pretreatment with R1-MVs suppresses the abnormally activated MAPKs (p38, ERK, and JNK) signals induced by H_2_O_2_ treatment. Therefore, it can be inferred that R1-MVs play a critical role in the protective mechanism via the inhibition of MAPK pathways in H_2_O_2_-exposed cell injury in HaCaT cells.

### R1-MVs stimulated the level of Nrf2 in H_2_O_2_ -induced oxidative stress in HaCaT cells

**Fig. 7:**
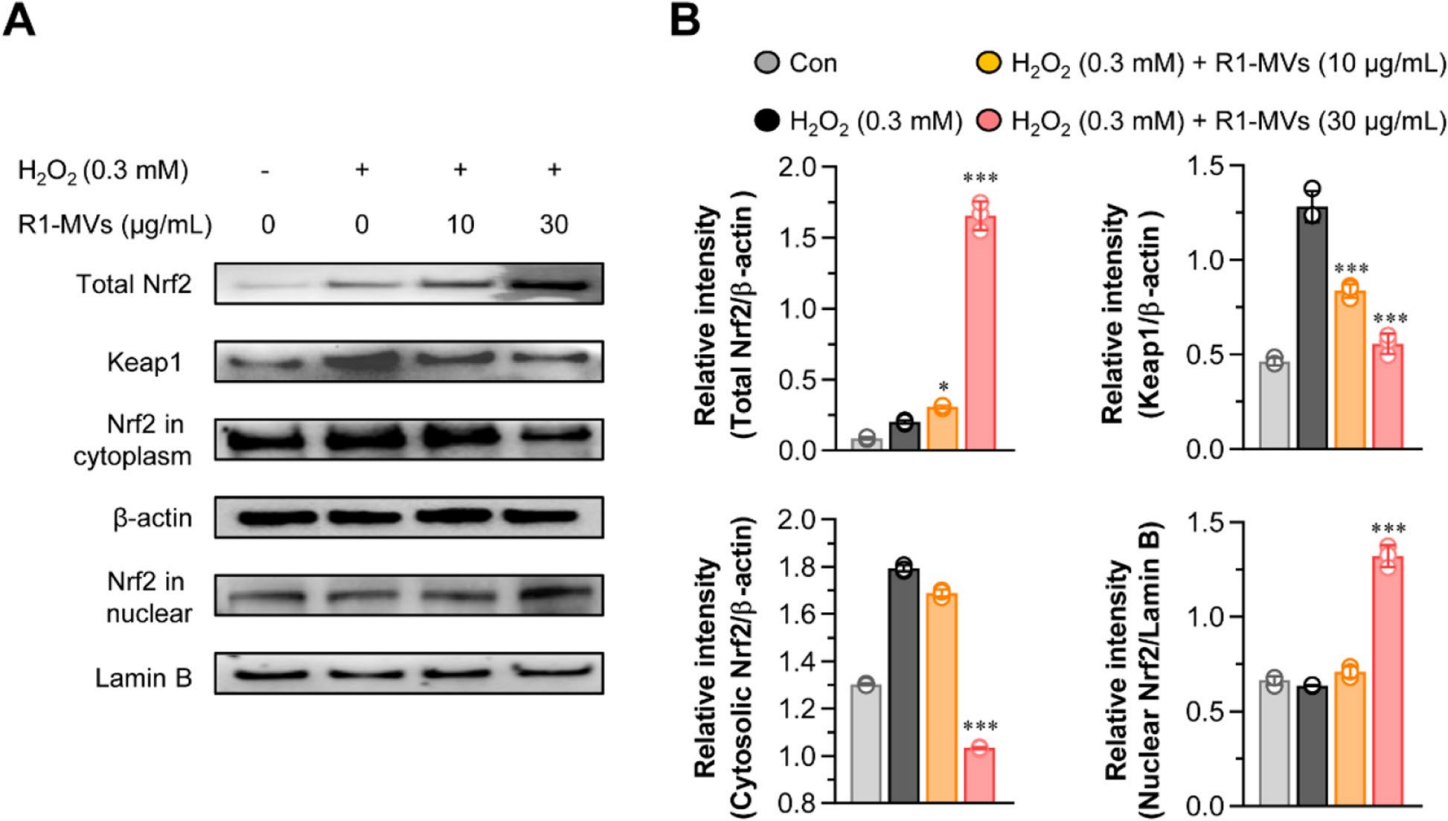
Effect of R1-MVs on the Nrf2/ARE pathway in H_2_O_2_-treated HaCaT cells. HaCaT cells were pre-processed with R1-MVs at different concentrations (10 and 30 μg/mL) for 12 h prior to exposure to H_2_O_2_ (0.3 mM) for 12 h. **A** The expression of Nrf2 and Keap1 in the cytoplasm and nucleus and the total levels of Nrf2 measured using western blotting analysis. **B** The relative Nrf2 and Keap1 band intensity of each protein evaluated using ImageJ software and expressed as a percentage. *p < 0.05 or ***p < 0.001 vs. H_2_O_2_-treated group. The data show the mean ± SD (*n* = 4 samples) of three representative experiments

Nrf2 is a transcription factor that plays an important role in the expression of phase II antioxidant enzymes, which are regulated by antioxidant response elements (ARE), to prevent oxidative stress-induced cell damage [33, 34]. Kelch-like ECH-associated protein 1 (Keap1) was identified as an NRF2 repressor that was up-regulated in response to oxidative stress, thereby inhibiting NRF2 [35]. To determine whether the Nrf2/ARE signaling pathway is involved in the oxidative protection ability of R1-MVs, the effect of R1-MVs on the protein level of Nrf2 in the cytoplasm and nucleus was assessed using western blotting. Pretreatment with R1-MVs (10 and 30 μg/mL) significantly enhanced the expression of Nrf2 in HaCaT cells exposed to H_2_O_2_. In addition, pretreatment with R1-MVs (10 and 30 μg/mL) decreased the protein level of Nrf2 and Keap1 in the cytoplasm of HaCaT cells exposed to H_2_O_2_. In contrast, Nrf2 expression in the nucleus was enhanced by pretreatment with R1-MVs (10 and 30 μg/mL) in HaCaT cells exposed to H_2_O_2_ (Fig. 7A and B). These results suggest that pretreatment with R1-MVs promote Nrf2 translocation from the cytoplasm to the nucleus, thereby attaining elevated binding ability to the downstream genes.

### MVs isolated from R1 DR2577 deletion mutant strain (ΔDR2577) are sensitive to H_2_O_2_ -induced oxidative stress compared to MVs isolated from R1 wild-type strain

**Fig. 8:**
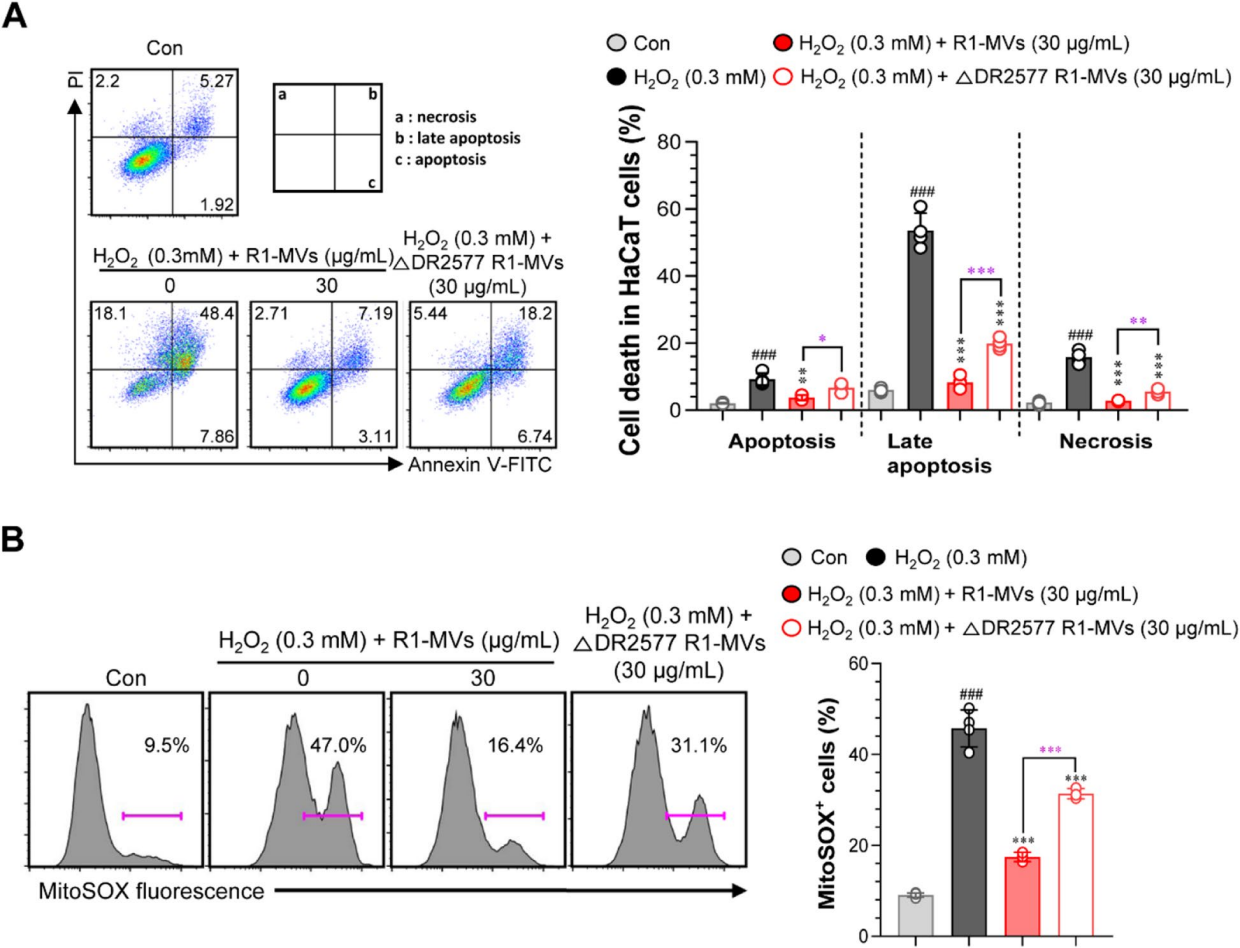
Effect of R1 DR2577 mutant strain (ΔDR2577) MVs isolated from ΔDR2577 (ΔDR2577 R1-MVs) against H_2_O_2_-induced oxidative stress. HaCaT cells were pre-processed with R1-MVs (30 μg/mL) or ΔDR2577 R1-MVs (30 μg/mL) for 12 h prior to exposure to H_2_O_2_ (0.3 mM) for 12 h. **A** Protective effect of MVs (R1 and ΔDR2577) against H_2_O_2_-induced oxidative stress in HaCaT cells assessed using annexin V/propidium iodide (PI) staining (PI^+^ cells, necrosis; AnnexinV^+^PI^+^ cells, late apoptosis; AnnexinV^+^ cells, early apoptosis). **B** Mitochondrial ROS scavenging effect of MVs (R1 and ΔDR2577) assessed using the Mitosox™ fluorescence probe in H_2_O_2_ exposed to HaCaT cells. ^###^*p* < 0.001 vs. control group; *p < 0.05, **p < 0.01, or ***p < 0.001 vs. H_2_O_2_-treated group; *p < 0.05, **p < 0.01, or ***p < 0.001 vs. H_2_O_2_/ΔDR2577 R1-MVs-treated group. The values show the mean ± SD (*n* = 4 samples) of three representative experiments

SlpA (DR2577) is a major S-layer component that binds to the carotenoid deinoxanthin, a powerful antioxidant molecule of the radioresistant bacterium *D. radiodurans* [36, 37]. Based on the protective effect of R1-MVs against H_2_O_2_-induced oxidative stress, we hypothesized that MVs isolated from ΔDR2577 (ΔDR2577 R1-MVs) would have a decreased protective effect against H_2_O_2_. We pretreated HaCaT cells with Δ DR2577 R1-MVs or R1-MVs followed by H_2_O_2_ treatment and assessed cell death using Annexin V/PI staining and MTT assay to confirm this hypothesis. The results indicated that pretreatment with R1-MVs (30 μg/mL) had a protective effect against H_2_O_2_-induced oxidative stress; however, the protective ability of ΔDR2577 R1-MVs (30 μg/mL) was significantly (necrosis; **p < 0.01, late apoptosis; ***p < 0.001, early apoptosis; *p < 0.05) lower than that of R1-MVs (Fig. 8A). In addition, to identify the mechanism underlying the protective effect of R1-MVs against H_2_O_2_ in HaCaT cells, MitoSOX-based flow cytometry was conducted to detect mitochondrial ROS levels. Interestingly, the ROS scavenging ability of ΔDR2577 R1-MVs (30 μg/mL) against H_2_O_2_-induced radical generation was notably (***p < 0.001) weaker than that of R1-MVs (30 μg/mL) (Fig. 8B). Taken together, considering the protective effect of R1-MVs against H_2_O_2_-induced oxidative stress, these results suggest that SlpA plays an important role in the protective mechanism against oxidative stress in R1-MVs.

## Discussion

In the present study, we successfully isolated and characterized MVs derived from *D. radiodurans*, and demonstrated that R1-MVs have antioxidant properties and ROS scavenging potential. Furthermore, we also demonstrated that R1-MVs protect against H_2_O_2_-induced oxidative damage in HaCaT cells. This protection may be related to the modulation of the MAPK and Nrf2/ARE signaling pathways and the mitochondrial apoptotic pathway associated with oxidative stress. Furthermore, by comparing the effect of R1-MVs with that of ΔDR2577 R1-MVs, we showed that the SlpA (DR2577) protein is important in regulating the protective roles of R1-MVs against H_2_O_2_-induced oxidative stress in HaCaT cells. To our knowledge, this is the first study to suggest that MVs derived from *D. radiodurans* exert protective effects against oxidative stress in keratinocytes.

Oxidative stress is a disturbance in the prooxidant-antioxidant balance in the body, which can disrupt redox signaling and control and/or cause molecular damage [38, 39]. Overproduction of aerobic metabolites, such as superoxide anion radicals, hydroxyl radicals, and H_2_O_2_, causes the pathogenesis of various diseases, such as metabolic and chronic disorders and cancers [40, 41]. H_2_O_2_ is one of the most common stimulators used to establish oxidative stress models [42]. It can diffuse throughout the mitochondria and cross cell membranes, generating excessive ROS, leading to lipid peroxidation and biomolecular denaturation [43]. MDA is a stable end-product of lipid peroxidation, a marker of oxidative stress [44]. SOD, CAT, and GSH are enzymatic and non-enzymatic antioxidant molecules that protect cells from radical attack and play important roles in antioxidant defense against oxidative stress [45–47]. Pretreatment with R1-MVs increased HaCaT cell viability, decreased the intracellular levels of ROS, enhanced the activities of enzymatic antioxidants (SOD and CAT) and GSH levels, and suppressed the release of MDA. These results indicated that R1-MVs could alleviate the cell damage induced by H_2_O_2_ via fortifying the antioxidant activities of HaCaT cells. In addition, mitochondria play a crucial role in cellular metabolism, signaling, and death pathways [48, 49]. ROS-mediated oxidative stress can induce mitochondrial damage, resulting in the reduced MMP and activation of mitochondrial apoptosis programs [50, 51]. This study showed that pretreatment with R1-MVs significantly inhibited the decrease in MMP in HaCaT cells exposed to H_2_O_2_. Furthermore, we assessed the anti-apoptotic effects of R1-MVs via a Bax/Bcl2-dependant pathway, which suggested that R1-MVs regulate the mitochondrial caspase-related apoptotic pathway.

The MAPK and Nrf2/ARE signaling pathways are associated with oxidative stress and antioxidant activities [52, 53]. Oxidative stress due to increased ROS production can activate MAPK signaling pathways via the activation of ERK, JNK, and p38 MAPK signaling proteins were involved in apoptosis via ROS generation [54–56]. In addition, as an antioxidant stress modulator, Nrf2 can be activated by excessive ROS exposure, and plays significant roles in the body’s antioxidant defenses [57]. Therefore, these two pathways can be used as an index factor when discovering new antioxidative substances [52, 58]. We found that R1-MVs suppressed the activation of ERK1/2, JNK, and p38 proteins induced by H_2_O_2_ in HaCaT cells. In addition, R1-MVs induced the accumulation of Nrf2 in the cell nucleus. These results indicate that R1-MVs may lead to Nrf2 translocation into the nucleus, which can promote binding to the ARE promoter and activate the transcription of antioxidant genes [59, 60]. Taken together, these results suggest that R1-MVs could regulate the Nrf2 pathway by promoting Nrf2 translocation from the cytoplasm to the nucleus thereby upregulating the Nrf2/ARE signaling pathway, which can subsequently restore the GSH homeostasis as well as increase the activities of CAT and SOD to suppress oxidative damage in HaCaT cells.

S-layers, which are external layers composed of a proteinaceous coat, play an important role in the protective mechanism of *D. radiodurans* against oxidative stress. Several studies have demonstrated that the S-layer deinoxanthin binding complex (SDBC) is resistant to UV and is thermostable, thereby playing a protective role in *D. radiodurans* [37, 61, 62]. This complex consists of the protein DR2577, a major surface layer constituent, and its cofactor deinoxanthin [37]. As the MVs reflect the characteristics of source cells [63], we hypothesized that MVs derived from R1 DR2577 (SlpA) mutant strain (DR2577 R1-MVs) have a weaker protective effect against H_2_O_2_-induced oxidative stress in HaCaT cells than R1-MVs. In the present study, we constructed a DR2577 mutant strain by deleting the SlpA protein and investigated the protective abilities of DR2577 R1-MVs compared to R1-MVs. The results showed that DR2577 R1-MVs decreased the cytoprotective and ROS scavenging effects against H_2_O_2_-induced oxidative stress in HaCaT cells compared to R1-MVs. These results suggest that SDBC, especially DR2577, plays an important role in regulating the protective mechanism of R1-MVs against oxidative stress.

Collectively, R1-MVs exerted a strong protective effect against H_2_O_2_-induced oxidative stress in HaCaT cells. Given that oxidative stress is associated with several complications, the antioxidation protection of R1-MVs was of considerable interest to medicine and public health. A comprehensive outlook on strategies involving R1-MVs for combating oxidative stress may open new avenues for novel therapeutics. Therefore, R1-MVs could be applied to ROS-mediated inflammatory diseases as well as the development of radioprotectors. However, the lack of omics analysis of bioactive molecules, such as proteins, lipids, and nucleic acids in R1-MVs is the limitation of our study. This analysis is needed to elucidate the specific molecules related to the antioxidative properties in R1-MVs. In future research, we will focus on conducting an omics study of R1-MVs. Furthermore, we have planned animal studies to investigate whether R1-MVs have potential as radioprotective materials in a total-body irradiation mouse model. These approaches might be anticipated because of the antioxidative properties of R1-MVs, as well as the therapeutic potential of MVs by themselves or as vehicles for the delivery of drug payload [64].

## Conclusions

In conclusion, we report for the first time the protective roles of MVs derived from *D. radiodurans* against H_2_O_2_-induced oxidative stress in HaCaT cells. The average diameter of R1-MVs, as analyzed by DLS, TEM, and SEM, was approximately 322 nm. Furthermore, we demonstrated that R1-MVs had a significant protective effect against H_2_O_2_-induced oxidative damage in HaCaT cells. These protective features may be related to the inhibition of the phosphorylation of MAPK signaling pathways to suppress mitochondrial dysfunction, as well as the activation of the Nrf2/ARE signaling pathway to increase antioxidant activity and decrease ROS generation in HaCaT cells. MVs derived from the R1 DR2577 mutant strain had a weaker protective effect against oxidative stress than those derived from wild-type R1, implying that SlpA protein plays a crucial role in R1-MVs against H_2_O_2_-induced oxidative stress.

## Material and methods

### Bacterial strain and culture conditions

*Deinococcus radiodurans* R1 (ATCC 13939) was obtained from the American Type Culture Collection (ATCC) and were cultured at 30 °C in tryptone glucose yeast extract (TGY) broth comprising 0.5% tryptone (Difco Laboratories, Detroit, MI, USA), 0.3% yeast extract (Difco Laboratories), and 0.1% glucose (Sigma–Aldrich, St. Louis, MO, USA) or on TGY plates with 1.5% Bacto-agar (Difco Laboratories). Antibiotics (8 μg/mL kanamycin; Sigma–Aldrich) were added to the medium, if necessary.

### Isolation and purification of R1-MVs

*Deinococcus radiodurans* strains were grown at 30 °C for 72 h under static conditions for isolation and purification of the R1-MVs. Briefly, after culturing in TGY broth for 72 h, the bacteria-free culture supernatants were harvested by centrifugation (10,000 × *g*, 30 min, 4 °C). The supernatant was filtered through a 0.45 μm bottle-top vacuum filter system (Corning, Merck KGaA, Darmstadt, Germany) using a Minimate™ tangential flow filtration (TFF) system with an Omega™ 300 K membrane capsule (Pall Scientific, NY, USA). The R1-EV pellets were harvested by ultracentrifugation (100,000 × *g*, 2 h, 4 °C), washed in sterile phosphate buffer saline (PBS; pH 7.4), and then purified by centrifugation using Optiprep density gradient medium (Sigma, #D1556, Steinheim, Germany). The R1-MVs were purified by ultracentrifugation (18 h, 170,000 × *g*, 4 °C; under no brake condition) in a discontinuous 60% Optiprep density gradient medium [step gradient ranging from 10 to 60% (w/v)]. The final MV pellet was resuspended in PBS and stored at − 80 °C. The protein content of R1-MVs was assessed using a bicinchoninic acid (BCA) protein assay kit (Thermo Scientific Pierce, Rockford, IL, USA) according to the manufacturer’s instructions.

### Characterization of R1-MVs

The hydrodynamic size of the R1-MVs was analyzed by dynamic light scattering (DLS) using a Zetasizer Nano ZS Zen3600 (Malvern, UK). For transmission electron microscopy (TEM), the samples were dispersed in ethanol, mounted onto a carbon support film on a 150-mesh nickel grid, and dried. For field-emission transmission electron microscopy (FE-TEM), the analysis was performed using a field-emission transmission electron microscope (JEM-2100F; JEOL Ltd., Japan) at an acceleration voltage of 200 kV. The samples were fixed with 3.7% glutaraldehyde (Sigma–Aldrich GmbH, Taufkirchen, Germany) in PBS for 15 min and used for scanning electron microscopy using an ESEM Quanta 400 scanning electron microscope (FEI, Hillsboro, Oregon, USA). After washing twice with PBS, the fixed samples were dehydrated using an ascending sequence of ethanol (40%, 60%, 80%, and 96–98%). After evaporation of ethanol, the samples were left to dry at room temperature (RT) for 24 h on a glass substrate and then analyzed by SEM after gold–palladium sputtering.

### Cell culture conditions

The immortalized human epidermal keratinocyte (HaCaT) cell line was obtained from Lonza and Korean Cell Line Bank (Seoul, Korea). The HaCaT cells were cultured in Dulbecco’s Modified Eagle’s Medium (DMEM; Biowest, Nuaille, France) supplemented with 10% fetal bovine serum (FBS, Biowest), and 1% penicillin and streptomycin (P/S, GIBCO, Carlsbad, CA, USA) at 37 °C in a humidified chamber with 5% CO_2_.

### 2-diphenyl-1-picrylhydrazyl (DPPH) radical scavenging assay

Assays using DPPH (Sigma-Aldrich) were carried out to investigate the free radical scavenging activity of R1-MVs. Briefly, 100 μL of the DPPH solution was added to 100 μL of R1-MVs (15.6, 31.3, 62.5, and 125 μg/mL). The mixture was incubated for 30 min at RT in the dark. DPPH solution is decolorized from deep violet to light yellow, upon receiving a hydrogen atom from an antioxidant sample. Absorbance was measured at 520 nm using a microplate reader (Biotek, Winooski, VT, USA). Vitamin C (300 μM) was used as a positive control. All measurements were performed in triplicate. The percent scavenging activity (%SA) was calculated using the following equation:

$$\mathrm{\%SA }= [({\mathrm{A}}_{\mathrm{sample}} - {\mathrm{A}}_{\mathrm{blank}})/{\mathrm{A}}_{\mathrm{blank}}] \times 100$$

### Ferric-reducing/antioxidant power (FRAP) assay

The FRAP assay was conducted as previously described [65]. The method is based on the reduction of a ferric 2,4,6-tripyridyl-s triazine complex (Fe3^+^-TPTZ) by antioxidants to the ferrous form (Fe2^+^-TPTZ). Briefly, the FRAP reagent (Sigma-Aldrich) comprising 10 mM TPTZ (ferrous iron) and 40 mM HCl was added to 300 mM sodium acetate buffer (pH 3.6) at 37 °C for 15 min. Reactions were started by adding 750 μL freshly prepared FRAP reagent to 50 μL R1-MVs. Vitamin C (300 μM) was used as a positive control. The absorbance was measured at 593 nm using a microplate reader (Biotek). All measurements were performed in triplicate.

### Effects of R1-MVs on cell viability of HaCaT keratinocytes

The viability of R1-MVs-treated HaCaT cells was assessed using the 3-(4,5-dimethylthiazol-2-yl)-2,5-diphenyltetrazolium bromide (MTT; Sigma-Aldrich) assay [66]. HaCaT cells were cultured at a density of 3 × 10^4^ cells/well in 96-well plates and incubated at 37 °C for 24 h. After culturing, the medium was discarded, and the cells were washed with PBS. The cells were treated with R1-MVs at concentrations of 1, 5, 10, 30, 50, and 100 μg/mL for 12 h. As a positive control, 0.5% DMSO was used. After culturing, the cells were washed, and MTT (0.5 mg/mL) was added to the wells at 37 °C for 4 h. Subsequently, the media was discarded, and 150 μL DMSO was added to each well to solubilize the formazan crystals. Formazan absorbance was analyzed at 540 nm using a microplate reader (Biotek). The viability of HaCaT cells is presented as a percentage of the control cell group.

### Effects of H_2_O_2_ on cell viability of HaCaT keratinocytes

To determine the optimal concentration of H_2_O_2_ that induced oxidative damage in vitro, HaCaT cells were treated with different concentrations of H_2_O_2_ (50, 100, 200, 300, 400, and 500 μM) for 12 h, and cell viability was measured using the MTT assay. Next, 300 μM H_2_O_2_ correspond to 70% cell viability and were chosen as optimal injury concentration.

### Determination of effect of R1-MVs on the viability of HaCaT cells under H_2_O_2_ -induced oxidative stress

To explore the protective effects of R1-MVs against oxidative stress, HaCaT cells were pretreated with nominal concentrations of R1-MVs (1, 5, 10, 30 and 50 μg/mL) for 12 h before H_2_O_2_-induced oxidative damage. The cells were then exposed to H_2_O_2_ (0.3 mM) for 12 h, and HaCaT cell viability was detected using the MTT method.

### Terminal deoxynucleotidyl transferase dUTP nick-end labeling (TUNEL) assay

HaCaT cells were seeded on glass slides for 24 h and then exposed to H_2_O_2_ (0.3 mM) in the presence or absence of R1-MVs for 12 h. The cells were fixed in 3.7% paraformaldehyde in 1 × PBS buffer for 30 min and were then permeabilized in 0.2% Triton X-100/PBS (Sigma, Germany) for 5 min. The glass slides were washed twice using PBS and 100 μL of the Equilibration Buffer was added for 10 min at 4 °C. Thereafter, the samples were cultured in 50 μL of TdT reaction mixture for 1 h at 37 °C in the dark. To stop the reaction, the glass slides were immersed in 2 × SSC for 15 min. Finally, DAPI nuclear stain was added along with a mounting medium and the samples were analyzed using a confocal laser scanning microscope (LSM510, Carl Zeiss, Jena, Germany).

### Determination of intracellular ROS contents in HaCaT cells

HaCaT cells were sequentially treated with R1-MVs (10 and 30 μg/mL) and H_2_O_2_ (0.3 mM) for 12 and 12 h, respectively. Subsequently, the supernatant was aspirated and cultured in an FBS-free medium containing dichlorodihydrofluorescein diacetate (DCFH-DA) (100 μM) for 30 min at 37 °C in the dark. Fluorescence intensity was analyzed using a confocal laser scanning microscope (LSM510), and quantitative analysis was performed using the ImageJ software. For flow cytometry analysis, the cells were detached by trypsinization (Trypsin–EDTA, Gibco, Paisley, UK) and resuspended in PBS. The fluorescence intensity of oxidized DCF was detected using a FACSVerse™ flow cytometer and FlowJo software.

### Measurement of the intracellular antioxidant molecules and malondialdehyde (MDA) levels

HaCaT cells were seeded at 1.0 × 10^7^ cells/well in a 100 mm dish for 24 h. After washing with serum-free media, the cells were treated with different concentrations of R1-MVs (5, 10, and 30 μg/mL) for 12 h. Then, H_2_O_2_ (0.3 mM) was added, and the cells were incubated at 37 °C for 12 h. A lysis buffer was used to resuspend the cells at 4 °C for 5 min after culturing, followed by centrifugation at 13,000 × *g* at 4 °C for 5 min to determine antioxidant molecule activities and MDA levels in the cell lysate. The activities of SOD, CAT, glutathione (GSH), and the level of MDA were measured using respective assay kits (BioVision, Milpitas, CA, USA).

### Detection of changes in the mitochondrial membrane potential (MMP)

MMP changes were measured using the JC-1 probe. JC-1 emits red fluorescence in non-apoptotic cells and green fluorescence in apoptotic or necrotic cells. Briefly, HaCaT cells were cultured on glass coverslips coated with poly-L-lysine (0.5 mg/mL)-coated glass coverslips for 12 h. After incubation, the cells were pretreated with R1-MVs (10 and 30 μg/mL) and exposed to H_2_O_2_ (0.3 mM) for 12 and 12 h, respectively. The medium was aspirated and incubated with 10 μg/mL of JC-1 (Thermo Fisher Scientific, Waltham, MA, USA) solution for 20 min at 37 °C in the dark, aspirated with the staining solution, and resuspended in the PBS. The fluorescence intensity was assessed using a confocal laser scanning microscope (LSM510). Red fluorescent JC-1 aggregates were detected by a 561 nm PE channel, while the monomeric green fluorescent form of JC-1 was detected by a 488 nm FITC channel. Quantification of JC-1 fluorescence was performed using the ImageJ software. The results are represented as a percentage of the control cells.

### Western blotting analysis

HaCaT cells were seeded in a 6-well plate and treated with H_2_O_2_ (0.3 mM) in the presence or absence of R1-MVs (10 and 30 μg/mL). Cytosolic and nuclear proteins were extracted using cell lysis buffer (RIPA buffer, Pierce, Rockford, IL, USA) and the CelLytic NuCLEAR Extraction Kit (Sigma-Aldrich), respectively. Protein concentration was measured using the BCA protein assay. Proteins were isolated using 10% SDS–PAGE and electrically transferred to polyvinylidene difluoride (PVDF) membranes. The membranes were blocked with 5% skim milk and incubated with the respective primary antibodies (1:1000 dilution; anti-Bcl-2, anti-Bax, anti-cytochrome C, anti-cleaved-caspase 3, anti-cleaved-caspase 8, anti-cleaved-caspase 9, anti-PARP, anti-p38, anti-ERK, anti-JNK, anti-p38, phosphorylated (p)-ERK, p-JNK, p-p38, Nrf2, β-actin, and anti-α-tubulin antibodies) overnight at 4 °C. Thereafter, the membranes were incubated with an HRP-conjugated secondary antibody (anti-rabbit Ab, 1:5000 dilution) for 1 h at RT. Proteins were visualized using an electrochemiluminescence advance kit (Millipore, Merck KGaA, Darmstadt, Germany). Primary and secondary antibodies were purchased from Cell Signaling Technology (Danvers, MA, USA) and Calbiochem (San Diego, CA, USA), respectively.

### ΔDR2577 deletion mutant construction

ΔDR2577 deletion mutants were constructed using the deletion mutagenesis method as previously described [67]. Briefly, polymerase chain reaction (PCR)-amplified fragments from the upstream and downstream regions of DR2577 were digested with the appropriate restriction enzymes and ligated into the corresponding sites of pKatAPH3. The recombinant plasmid was then transformed into *D. radiodurans* cells. The mutant strains were selected on TGY agar plates (0.5% tryptone, 0.3% yeast extract, and 0.1% glucose) supplemented with 8 μg/mL kanamycin (Sigma-Aldrich). All constructs were confirmed using diagnostic PCR and nucleotide sequencing. The primers used in this study are listed in Table S1.

### Annexin V and propidium iodide (PI) staining

To investigate apoptosis, HaCaT cells were treated with 30 μg/mL of R1-MVs or ΔDR2577-R1-MVs for 12 h prior to treatment with H_2_O_2_ (0.3 mM) for 12 h at 37 °C and were analyzed using Annexin V/PI staining (BD Bioscience, San Jose, CA, USA). Cells were harvested and stained with annexin V (1:50 dilution with annexin V binding buffer, BD Bioscience, San Jose, CA, USA) for 15 min at RT. After washing with annexin V binding buffer, cells were stained with PI (1:25 dilution with annexin V binding buffer) for 10 min at RT. Necrotic, late apoptotic, and apoptotic cell death were assessed by analyzing cells positive for Annexin V, PI, or both, respectively, using a FACSverse cytometer and FlowJo software (V10, BD Bioscience).

### Determination of mitochondrial ROS contents in HaCaT cells

The generation of ROS by mitochondria was analyzed using the MitoSOX mitochondrial superoxide indicator (Thermo Fisher Scientific). HaCaT cells were incubated with R1-MVs (30 μg/mL) or ΔDR2577-R1-MVs (30 μg/mL) for 12 h prior to treatment with H_2_O_2_ (0.3 mM) for 12 h at 37 °C The HaCaT cells were cultured with 5 μM MitoSOX reagent for 10 min at 37 °C in the dark, washed, and resuspended in PBS. The samples were analyzed using a FACSverse cytometer and FlowJo software (V10, BD Biosciences).

### Statistical analysis

Statistical analyses were performed using Tukey’s multiple comparison test or an unpaired t-test using GraphPad Prism 7 (2018, GraphPad, San Diego, CA, USA). Data are expressed as the mean ± SD. P-values of < 0.05 were considered statistically significant.

## Supplementary Information

**Additional file 1:** **Supplementary Table 1.** Primers in this study.

**Additional file 2:** **Supplementary Figure S1.**Characterization of ΔDR2577 R1-EVs by DLS and SEM. (A) Size distribution of EVs was assessed by dynamic light scattering (DLS) analysis. (B) Morphology of EVs were visualized by scanning electron microscopy (SEM). Scale bar = 200 nm.

**Additional file 3:** **Supplementary Figure S2.** Growth curve of *D. radiodurans*. (A) *D. radiodurans* were grown in TGY medium and optical density (OD 600) measurements were employed to estimate the growth of *D. radiodurans.*

**Additional file 4:** **Supplementary Figure S3. **Yield and NTA analysis of membrane vesicles derived from *D. radiodurans*. (A) *Y*ield of R1-MVs in the various culture points. Protein concentration of R1-MVs were measured by the BCA assay. (B) R1-MVs were identified by the Nanoparticle tracking analysis (NTA). R1-MVs has 320 nm with the 3.9 × 10^9^ particles/mL in 0.26 μg/mL of R1-MVs.

**Additional file 5:** **Supplementary Figure S4. **Uptake of CFSE-labelled R1-MVs inside the HaCaT cells.(A) HaCaT cells were treated with CFSE-labeled R1-MVs for indicated time periods and uptake levels (CFSE ^+^ cells) of CFSE-labeled MVs were analyzed by flow cytometry (*n* = 3 per time periods).

## Data Availability

Not applicable.

## Abbreviations

ARE: Antioxidant response element
BCA: Bicinchoninic acid
CAT: Catalase
DCFH-DA: Dichlorodihydrofluorescein diacetate
DdrB: DNA damage response protein B
DdrO: DNA damage response protein O
DLS: Dynamic light scattering
DMEM: Dulbecco’s modified eagle’s medium
DPPH: 2,2-Diphenyl-1-picrylhydrazyl
EVs: Extracellular vesicles
FBS: Fetal bovine serum
FE-TEM: Field-emission transmission electron microscopy
Fe2^+^-TPTZ: Ferrous,4,6-tripyridyl-s triazine complex
Fe3^+^-TPTZ: Ferric 2,4,6-tripyridyl-s triazine complex
FRAP: Ferric-reducing/antioxidant power
GSH: Glutathione
GSSG: Oxidized glutathione
IrrE: Inducible response regulator E
MAPK: Mitogen-activated protein kinase
MDA: Malondialdehyde
MMP: Mitochondrial membrane potential
MTT: 3-4,5-Dimethylthiazol-2-yl-2,5-diphenyltetrazolium bromide
MV: Membrane vesicles
Nrf2: Nuclear factor E2-related factor 2
P/S: Penicillin and streptomycin
PBS: Phosphate buffer saline
PCR: Polymerase chain reaction
PI: Propidium iodide
PVDF: Polyvinylidene difluoride
R1-MVs: *D. radiodurans*-derived MVs
ROS: Reactive oxygen species
RT: Room temperature
SDBC: S-layer deinoxanthin binding complex
SEM: Scanning electron microscopy
SOD: Superoxide dismutase
TEM: Transmission electron microscopy
TFF: Tangential flow filtration
TGY: Tryptone glucose yeast extract
TUNEL: Terminal deoxynucleotidyl transferase dUTP nick-end labeling
