# Supplementary material for: Deinococcus radiodurans-derived membrane vesicles protect HaCaT cells against H2O2-induced oxidative stress via modulation of MAPK and Nrf2/ARE pathways
Source: Biol Proced Online. 2023 Jun 16;25:17. doi: 10.1186/s12575-023-00211-4 (PMC10273539; doi:10.1186/s12575-023-00211-4)
Supplement: Supplementary file 1 — Additional file 1: Supplementary Table 1. Primers in this study. [file 12575_2023_211_MOESM1_ESM.docx]

Supplementary Table 1. Primers in this study

| **Primer** | **Sequence (5’ to 3’)** | **Restriction enzyme** |
| --- | --- | --- |
| DR2577 upF | CTCGGTACCGTCACCACACCTATGTTT | *KpnⅠ* |
| DR2577 upR | TTCGAT ATCTTAAATTTCGGACTGGG | *EcoRⅤ* |
| DR2577 downF | GACCTGCAGAGCCCCTAGTTCCCGGCATT | *PtsⅠ* |
| DR2577 downR | TTGCATGCGTAAATAGGGAGACGAGG | *SphⅠ* |
