# Supplementary material for: Deinococcus radiodurans-derived membrane vesicles protect HaCaT cells against H2O2-induced oxidative stress via modulation of MAPK and Nrf2/ARE pathways
Source: Biol Proced Online. 2023 Jun 16;25:17. doi: 10.1186/s12575-023-00211-4 (PMC10273539; doi:10.1186/s12575-023-00211-4)
Supplement: Supplementary file 2 — Additional file 2: Supplementary Figure S1.Characterization of ΔDR2577 R1-EVs by DLS and SEM. (A) Size distribution of EVs was assessed by dynamic light scattering (DLS) analysis. (B) Morphology of EVs were visualized by scanning electron microscopy (SEM). Scale bar = 200 nm. [file 12575_2023_211_MOESM2_ESM.docx]

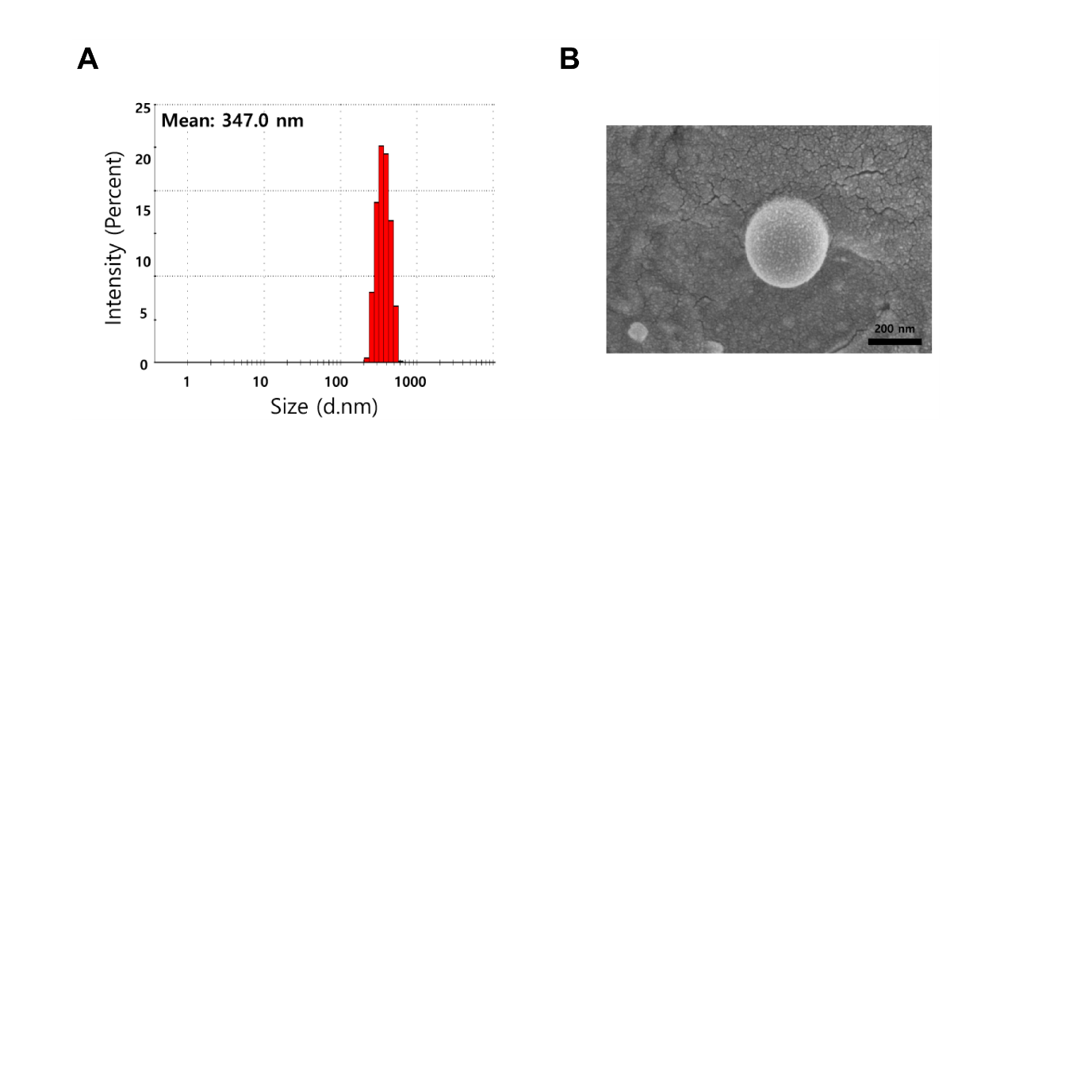


**Supplementary Figure S1. Characterization of ΔDR2577 R1-EVs by DLS and SEM.** (A) Size distribution of EVs was assessed by dynamic light scattering (DLS) analysis. (B) Morphology of EVs were visualized by scanning electron microscopy (SEM). Scale bar = 200 nm.
