# Supplementary material for: Deinococcus radiodurans-derived membrane vesicles protect HaCaT cells against H2O2-induced oxidative stress via modulation of MAPK and Nrf2/ARE pathways
Source: Biol Proced Online. 2023 Jun 16;25:17. doi: 10.1186/s12575-023-00211-4 (PMC10273539; doi:10.1186/s12575-023-00211-4)
Supplement: Supplementary file 3 — Additional file 3: Supplementary Figure S2. Growth curve of D. radiodurans. (A) D. radiodurans were grown in TGY medium and optical density (OD 600) measurements were employed to estimate the growth of D. radiodurans. [file 12575_2023_211_MOESM3_ESM.docx]

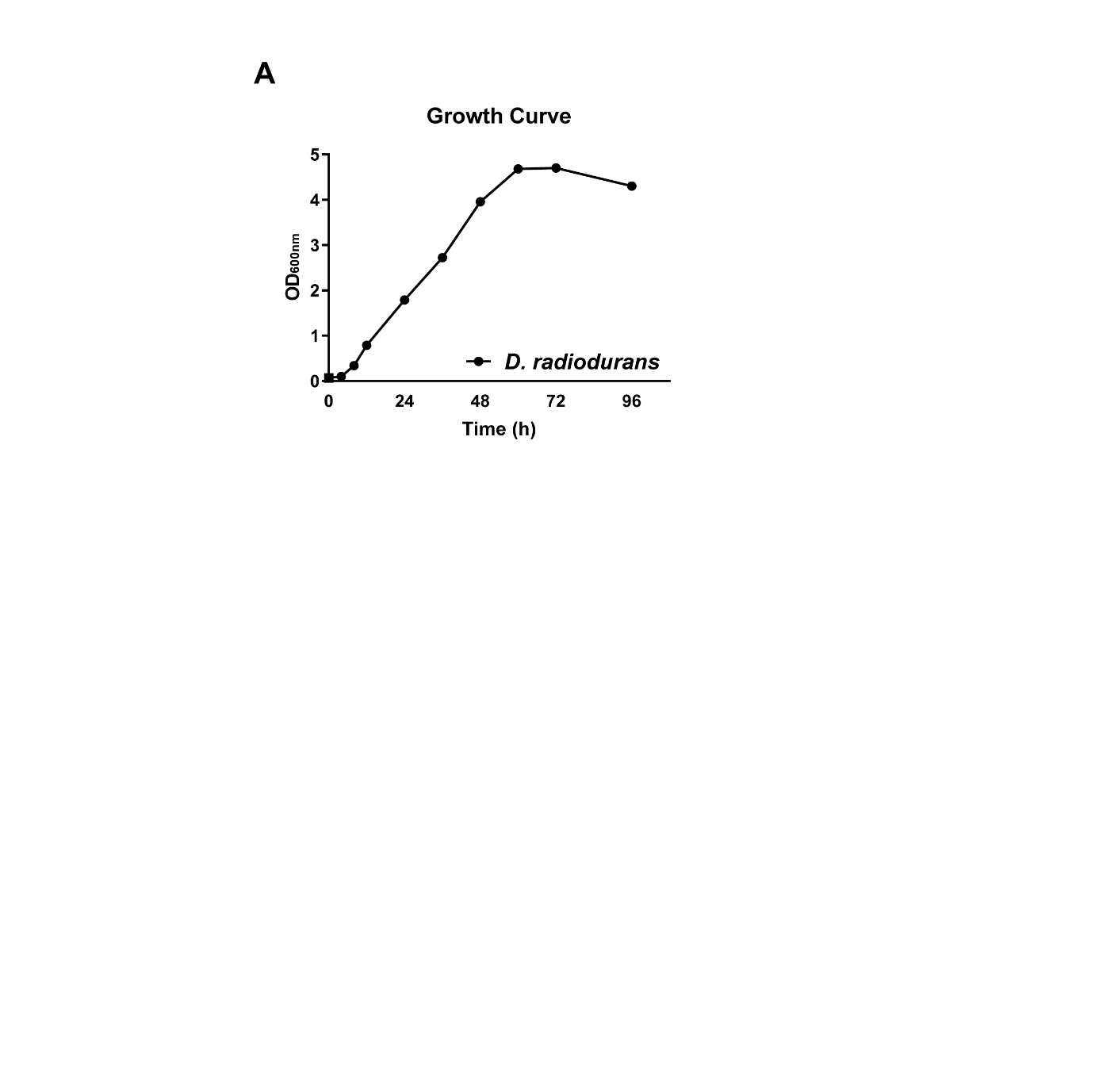


**Supplementary Figure S2. Growth curve of *D. radiodurans*.** (A) *D. radiodurans* were grown in TGY medium and optical density (OD 600) measurements were employed to estimate the growth of *D. radiodurans.*
