# Supplementary material for: Deinococcus radiodurans-derived membrane vesicles protect HaCaT cells against H2O2-induced oxidative stress via modulation of MAPK and Nrf2/ARE pathways
Source: Biol Proced Online. 2023 Jun 16;25:17. doi: 10.1186/s12575-023-00211-4 (PMC10273539; doi:10.1186/s12575-023-00211-4)
Supplement: Supplementary file 4 — Additional file 4: Supplementary Figure S3. Yield and NTA analysis of membrane vesicles derived from D. radiodurans. (A) Yield of R1-MVs in the various culture points. Protein concentration of R1-MVs were measured by the BCA assay. (B) R1-MVs were identified by the Nanoparticle tracking analysis (NTA). R1-MVs has 320 nm with the 3.9 × 10^9^ particles/mL in 0.26 μg/mL of R1-MVs. [file 12575_2023_211_MOESM4_ESM.docx]

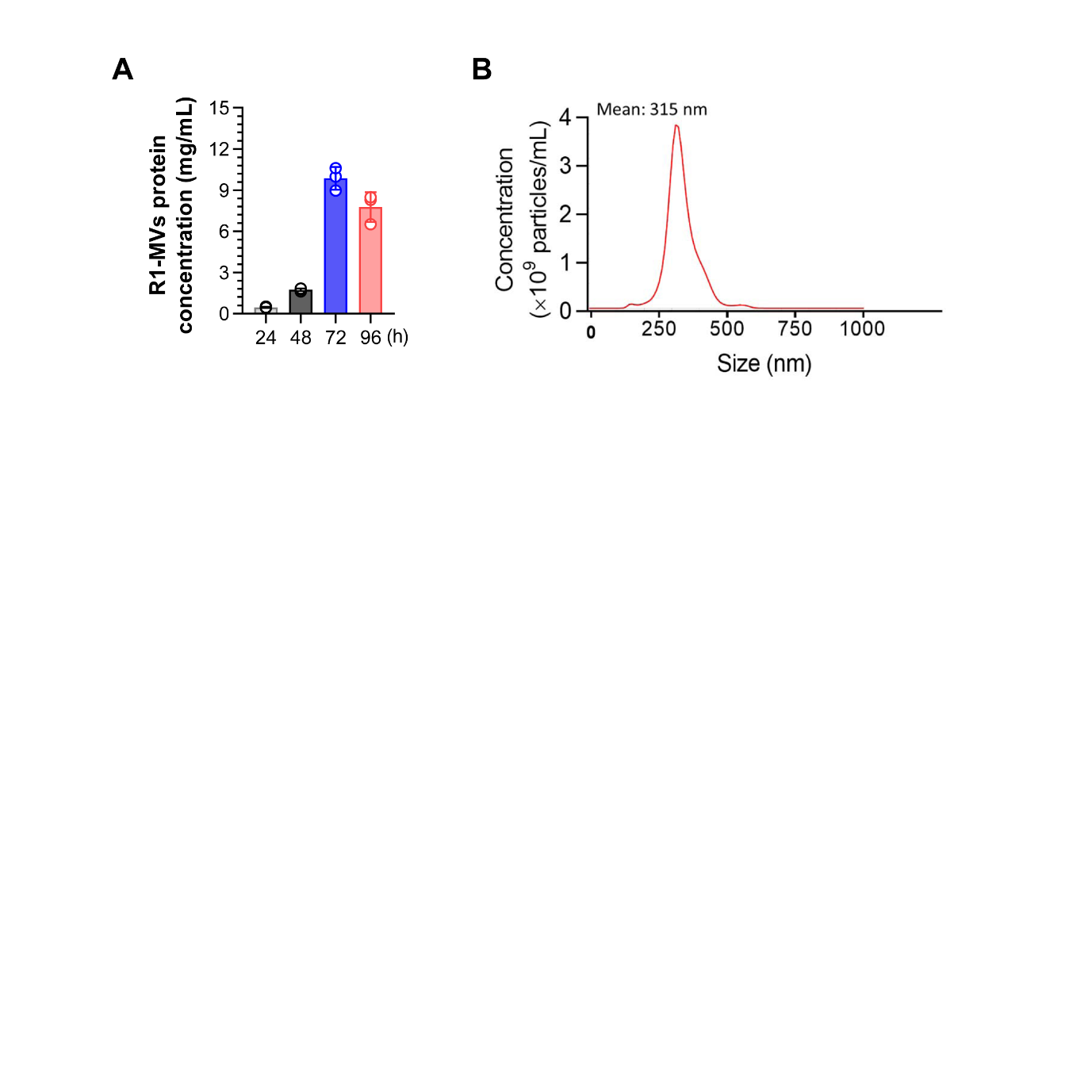


**Supplementary Figure S3. Yield and NTA analysis of membrane vesicles derived from *D. radiodurans*.** (A) *Yield of R1-MVs in the various culture points. Protein concentration of R1-MVs were measured by the BCA assay. (B) R1-MVs were identified by the Nanoparticle tracking analysis (NTA). R1-MVs has 320 nm with the 3.9 × 10^9^ particles/mL in 0.26 μg/mL of R1-MVs*
