# Supplementary material for: Deinococcus radiodurans-derived membrane vesicles protect HaCaT cells against H2O2-induced oxidative stress via modulation of MAPK and Nrf2/ARE pathways
Source: Biol Proced Online. 2023 Jun 16;25:17. doi: 10.1186/s12575-023-00211-4 (PMC10273539; doi:10.1186/s12575-023-00211-4)
Supplement: Supplementary file 5 — Additional file 5: Supplementary Figure S4. Uptake of CFSE-labelled R1-MVs inside the HaCaT cells.(A) HaCaT cells were treated with CFSE-labeled R1-MVs for indicated time periods and uptake levels (CFSE + cells) of CFSE-labeled MVs were analyzed by flow cytometry (n = 3 per time periods). [file 12575_2023_211_MOESM5_ESM.docx]

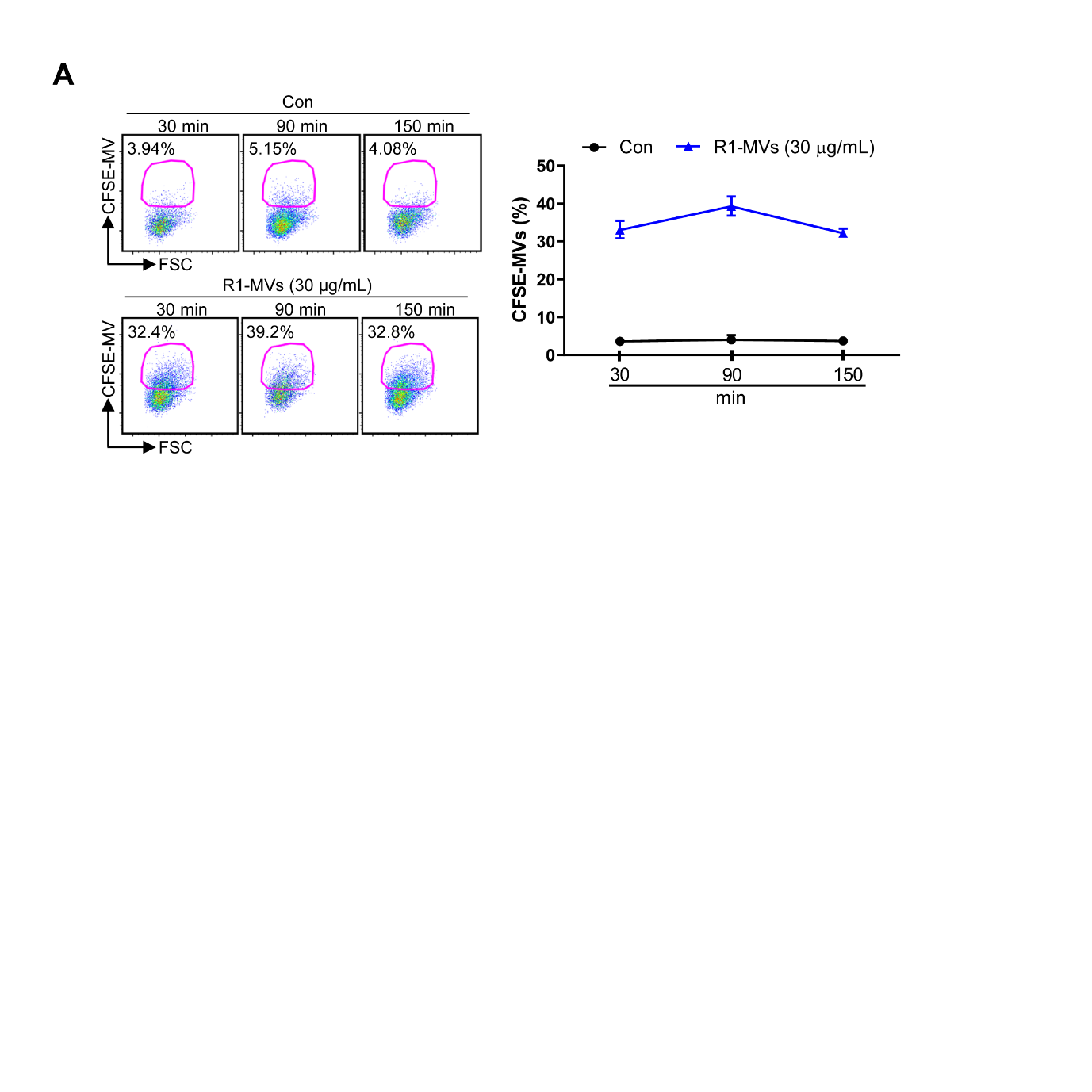


***Supplementary Figure S4. Uptake of CFSE-labelled R1-MVs inside the HaCaT cells.*** *(A) HaCaT cells were treated with CFSE-labeled R1-MVs for indicated time periods and uptake levels (CFSE ^+^ cells) of CFSE-labeled MVs were analyzed by flow cytometry (n = 3 per time periods).*
